# Supplementary material for: Blocking D2/D3 dopamine receptors in male participants increases volatility of beliefs when learning to trust others
Source: Nat Commun. 2023 Jul 8;14:4049. doi: 10.1038/s41467-023-39823-5 (PMC10329681; doi:10.1038/s41467-023-39823-5)
Supplement: Supplementary file 1 — Supplementary Information [file 41467_2023_39823_MOESM1_ESM.pdf]

Supplementary Materials for

Blocking D2/D3 dopamine receptors in male participants increases volatility of beliefs when learning to trust others

Mikus, Nace [1, 2], Eisenegger, Christoph [1, 3,*], Mathys, Chris [2, 4, 5], Clark, Luke [6, 7], Müller, Ulrich [3, 8], Robbins, Trevor W. [3], Lamm, Claus [1, +], Naef, Michael [9, +]

[+] These authors jointly supervised this work: Lamm, Claus, Naef, Michael

[*] Correspondence to: Nace Mikus, nace.mikus@univie.ac.at, Department of Cognition, Emotion, and Methods in Psychology, Faculty of Psychology, University of Vienna, Liebiggasse 5, 1010 Vienna,

This pdf file includes:

- Supplementary Methods
    - Supplementary Tables
    - Side effects
- Supplementary Notes 1&2
- Supplementary Figures

[*] Deceased on 27th February 2017

Supplementary Methods

Supplementary Tables

Abs_change~Treatment*Trial + (1|ID)	Estimate	Est.Error	Q2.5	Q97.5
**Intercept**	-0.15	0.076	-0.295	-0.003
**TreatmentSulpiride**	0.309	0.106	0.108	0.518
**Trial**	-0.015	0.003	-0.021	-0.009
**TreatmentSulpride:Trial**	0.007	0.004	-0.001	0.015
Abs_change~Treatment*Trial + (Trial|ID)				
**Intercept**	-0.114	0.069	-0.246	0.025
**Treatmentsulpiride**	0.234	0.097	0.043	0.427
**Trial_c**	-0.012	0.005	-0.022	-0.001
**Treatmentsulpiride:Trial_c**	0.007	0.007	-0.008	0.022

Supplementary Table 1: Results of the model Bayesian mixed model predicting absolute changes in investment from one trial to the next including drug treatment and trial as predictors. Similar results are achieved when the slope for the trial is allowed to vary per participant. Treatment (coded as 1 sulpiride and 0 placebo), trial was used as a continuous variable and centred. Refer to the code about the effect in native space and the calculation of effect sizes.

Abs_change ~Treatment*Trial + (1|ID)	Value	Std.Error	DF	t-value	p-value
**(Intercept)**	-0.113	0.066	3570	-1.715	0.086
**TreatmentSulpiride**	0.235	0.093	74	2.515	0.014
**Trial**	-0.012	0.003	3570	-3.87	<10e3
**TreatmentSulpiride:Trial**	0.007	0.004	3570	1.626	0.104

Supplementary Table 2: The output of the non-Bayesian absolute change model. T-values all two-sided, p values uncorrected.

log(1+Abs_change) ~ Treatment*Trial + (1|ID)	Value	Std.Error	DF	t-value	p-value
**(Intercept)**	-0.149	0.073	3570	-2.045	0.041
**Treatmentsulpiride**	0.309	0.103	74	3.004	0.004
**Trial**	-0.015	0.003	3570	-5.084	<10e3
**Treatmentsulpiride:Trial**	0.007	0.004	3570	1.715	0.086

Supplementary Table 3: The non-Bayesian absolute change model reran again with a transformed outcome variable. Because the absolute change variable is positive, we use a log transform of a translation. T-values all two-sided, p values uncorrected.

Abs_change ~ Treatment*Genotype *Trustee *Trial + (Trustee |ID)	Estimate	Est.Error	Q2.5	Q97.5
**Intercept**	-0.109	0.069	-0.25	0.024
**Treatmentsulpride**	0.234	0.099	0.044	0.431
**Trial**	-0.013	0.003	-0.018	-0.007
**Trustee**	0.003	0.07	-0.133	0.143
**Genotype**	0.079	0.138	-0.195	0.348
**Treatmentsulpride:Trial**	0.007	0.004	-0.001	0.016
**Treatmentsulpride:Trustee**	-0.145	0.1	-0.339	0.046
**Trial:Trustee**	0.015	0.006	0.003	0.027
**Treatmentsulpride:Genotype**	-0.111	0.198	-0.494	0.278
**Trial:Genotype**	-0.011	0.006	-0.023	0
**Trustee:Genotype**	-0.002	0.137	-0.272	0.274
**Treatmentsulpride:Trial:Trustee**	-0.007	0.009	-0.024	0.01
**Treatmentsulpride:Trial:Genotype**	0.021	0.009	0.004	0.038
**Treatmentsulpride:Trustee:Genotype**	-0.051	0.194	-0.433	0.332
**Trial:Trustee:Genotype**	-0.016	0.012	-0.04	0.008
**Treatmentsulpride:Trial:Trustee:Genotype**	0.042	0.017	0.009	0.076

Supplementary Table 4: Bayesian mixed model predicting absolute changes in investment from one trial to the next including drug treatment, trial, genotype and trustee as predictors. Treatment coded as 1 sulpiride and 0 placebo, trial used as a continuous variable. Variables Trial, Genotype (0.5 A1+, -0.5 A1-) and Trustee (0.5 good, -0.5 bad) were centred**.**

Change ~Treatment*Genotype*Backtransfer*Trustee+ (Trustee |ID)	Value	Std.Error	DF	t-value	p-value
**(Intercept)**	0.017	0.038	3560	0.436	0.663
**Treatmentsulpiride**	-0.002	0.051	72	-0.034	0.973
**Backtransfer**	0.204	0.038	3560	5.337	<10e3
**GenotypeA1-**	-0.033	0.051	72	-0.65	0.518
**Trustee**	-0.105	0.076	3560	-1.375	0.169
**Treatmentsulpiride:Backtransfer**	0.114	0.051	3560	2.218	0.027
**Treatmentsulpiride:GenotypeA1-**	0.03	0.072	72	0.42	0.676
**Backtransfer:GenotypeA1-**	0	0.051	3560	-0.004	0.997
**Treatmentsulpiride:Trustee**	-0.078	0.102	3560	-0.759	0.448
**Backtransfer:Trustee**	-0.027	0.076	3560	-0.352	0.725
**GenotypeA1-:Trustee**	-0.012	0.103	3560	-0.112	0.911
**Treatmentsulpiride:Backtransfer:GenotypeA1-**	-0.171	0.072	3560	-2.363	0.018
**Treatmentsulpiride:Backtransfer:Trustee**	-0.084	0.102	3560	-0.819	0.413
**Treatmentsulpiride:GenotypeA1-:Trustee**	0.145	0.145	3560	1.003	0.316
**Backtransfer:GenotypeA1-:Trustee**	0.094	0.103	3560	0.917	0.359
**Treatmentsulpiride:Backtransfer:GenotypeA1-:Trustee**	-0.002	0.145	3560	-0.013	0.99

Supplementary Table 5 A frequentist mixed model predicting changes in investment from one trial to the next including drug treatment, trial, genotype, back-trasnfer and trustee as predictors. Treatment coded as 1 sulpiride and 0 placebo, trial used as a continuous variable. Variables Trial, Genotype (0.5 A1+, -0.5 A1-), Backtransfer (-1 betray, 1 equalize) and Trustee (0.5 good, -0.5 bad) were centred**.** T-values all two-sided, p values uncorrected.

ReciprocalTrial ~Treatment*Genotype*Trustee_c + (Trustee |ID), family = binomial	Estimate	Est.Error	Q2.5	Q97.5
**Intercept**	0.634	0.133	0.377	0.894
**Treatmentsulpiride**	0.399	0.179	0.045	0.755
**GenotypeA1-**	-0.015	0.179	-0.37	0.335
**Trustee**	-0.027	0.23	-0.474	0.427
**Treatmentsulpiride:GenotypeA1-**	-0.223	0.256	-0.727	0.276
**Treatmentsulpiride:Trustee**	-0.023	0.315	-0.637	0.591
**GenotypeA1-:Trustee**	0.375	0.307	-0.219	0.975
**Treatmentsulpiride:GenotypeA1-:Trustee**	-0.103	0.439	-0.965	0.76

Supplementary Table 6: Bayesian mixed logistic model predicting Reciprocal trials from drug treatment, genotype and trustee as predictors.

ReciprocalTrial ~Treatment*Genotype*Trustee_c + (Trustee_c|ID), family = binomial	Estimate	Std. Error	z value	Pr(>|z|)
**(Intercept)**	0.604	0.126	4.804	<10e3
**Treatmentsulpiride**	0.392	0.171	2.296	0.022
**GenotypeA1-**	-0.018	0.169	-0.106	0.916
**Trustee**	-0.049	0.22	-0.222	0.824
**Treatmentsulpiride:GenotypeA1-**	-0.217	0.24	-0.903	0.366
**Treatmentsulpiride:Trustee**	-0.009	0.299	-0.03	0.976
**GenotypeA1-:Trustee**	0.38	0.295	1.289	0.197
**Treatmentsulpiride:GenotypeA1-:Trustee**	-0.108	0.421	-0.257	0.797

Supplementary Table 7: Frequentist model mixed logistic model predicting Reciprocal trials from drug treatment, genotype and trustee as predictors. P values uncorrected.

ReciprocalTrial ~log(Serum)*Genotype*Trustee_c + (Trustee |ID), family = binomial, data = Sulpiride group only	Estimate	Est.Error	Q2.5	Q97.5
**Intercept**	1.012	0.116	0.783	1.236
**logserum_s**	0.186	0.113	-0.04	0.41
**GenotypeA1-**	-0.235	0.17	-0.568	0.103
**Trustee**	-0.058	0.175	-0.404	0.282
**logserum_s:GenotypeA1-**	-0.222	0.175	-0.565	0.126
**logserum_s:Trustee**	0.325	0.169	-0.003	0.661
**GenotypeA1-:Trustee**	0.263	0.256	-0.241	0.774
**logserum_s:GenotypeA1-:Trustee**	-0.429	0.256	-0.935	0.074

Supplementary Table 8: Bayesian mixed logistic model predicting Reciprocal trials Sulpiride group only, from serum levels (log-scaled), genotype and trustee as predictors.

MistakeTrial ~Logserum*Genotype*Trustee_c + (Trustee |ID), family = binomial, data = Sulpiride group only	Estimate	Est.Error	Q2.5	Q97.5
**Intercept**	-2.068	0.248	-2.589	-1.607
**logserum_s**	-0.331	0.242	-0.818	0.143
**GenotypeA1-**	0.427	0.361	-0.314	1.136
**Trustee**	-0.156	0.233	-0.622	0.300
**logserum_s:GenotypeA1-**	0.941	0.356	0.25	1.663
**logserum_s:Trustee**	-0.57	0.231	-1.048	-0.127
**GenotypeA1-:Trustee**	-0.191	0.314	-0.812	0.428
**logserum_s:GenotypeA1-:Trustee**	0.595	0.315	-0.01	1.227

Supplementary Table 9: Bayesian mixed logistic model predicting Mistake trials Sulpiride group only, from serum levels (log-scaled), genotype and trustee as predictors.

Punishment ~ Treatment*Genotype + (1|ID)	Estimate	Est.Error	Q2.5	Q97.5
**Intercept**	6.16	1.177	3.893	8.447
**Treatmentsulpride**	1.776	1.446	-1.097	4.619
**genotypeA1-**	-0.764	1.435	-3.585	2.086
**Treatmentsulpride:genotypeA1-**	-0.476	1.83	-4.091	3.077

Supplementary Table 10: Negative reciprocity task: Bayesian mixed model predicting Punishment from treatment, genotype, and trustee as predictors.

Back-transfer ~ Treatment*Genotype + (1|ID)	Estimate	Est.Error	Q2.5	Q97.5
**Intercept**	380.565	14.042	349.795	403.707
**Treatmentsulpride**	-0.016	2.923	-5.911	5.634
**genotypeA1-**	-0.219	2.978	-5.983	5.698
**Treatmentsulpride:genotypeA1-**	-0.095	3	-5.999	5.792

Supplementary Table 11: Positive reciprocity task: Bayesian mixed model predicting Back-transfer from treatment, genotype and trustee as predictors.

PrecisionWeights_s ~ logserum_s * Genotype + (1 | ID)	Estimate	Est.Error	Q2.5	Q97.5
**Intercept**	0.489	0.137	0.219	0.762
**logserum_s**	0.286	0.126	0.037	0.539
**GenotypeA1-**	-1.118	0.2	-1.507	-0.721
**logserum_s:GenotypeA1-**	-0.218	0.193	-0.595	0.158

Supplementary Table 12: Bayesian mixed model predicting PrecisionWeights from log serum levels and genotype in the Sulpiride group only.

prec_weights_s ~ logserum_s * Genotype + (1 | ID)	Value	Std.Error	DF	t-value	p-value
**(Intercept)**	0.511	0.13	1862	3.94	<10e3
**logserum_s**	0.293	0.126	34	2.317	0.027
**GenotypeA1-**	-1.165	0.194	34	-6.005	<10e3
**logserum_s:GenotypeA1-**	-0.234	0.196	34	-1.196	0.24

Supplementary Table 13: Frequentist mixed model predicting precision weights from log serum levels and genotype in the Sulpiride group only. T-values all two-sided, p values uncorrected.

Points Earned ~ Treatment*Genotype*Trustee	Estimate	Est.Error	Q2.5	Q97.5
**Intercept**	324.303	7.176	309.962	338.305
**TrusteeBad**	-120.505	10.308	-140.018	-100.036
**Treatmentsulpiride**	7.333	9.678	-12.076	26.262
**GenotypeA1-**	6.415	9.543	-11.988	25.366
**TrusteeBad:Treatmentsulpiride**	-1.634	14.08	-29.781	26.353
**TrusteeBad:GenotypeA1-**	-12.337	13.671	-40.087	13.72
**Treatmentsulpiride:GenotypeA1-**	-8.555	13.507	-34.12	19.582
**TrusteeBad:Treatmentsulpiride:GenotypeA1-**	17.192	19.632	-21.076	55.809

Supplementary Table 13: Bayesian linear model predicting overall points earned in the repeated trust game from Genotype, Treatment and Trustee.

Side Effects

The effect of sulpiride on blood-pressure and heart-rate, self-reported side-effects and mood were adopted from previous published work with the same cohort[1,2]. Supplementary Table 13 shows all side-effects measures, their changes over time, as well as the results of a Mann-Whitney test for differences across treatment groups. Significance levels are not above chance level if corrected for multiple testing (Holm-Bonferroni correction).

side effects	time point	N	Plac.	Sulp.	Sign. (*p*)
Heart rate	base	76	69.2	67.5	0.807
	3 h	76	63.8	64.9	0.596
	δ	76	-5.4	-2.6	0.666
Blood pressure systolic [mm hg]	base	76	132.2	132.8	0.783
	3 h	76	128.1	127.5	0.975
	δ	76	-4.1	-5.4	0.621
Blood pressure diastolic [mm hg]	base	76	76.1	76.9	0.856
	3 h	76	72.0	70.9	0.629
	δ	76	-4.1	-6.0	0.240
VAS: alertness (mean)	base	76	22.6	23.4	0.880
	3 h	75	28.5	28.8	0.945
	δ	75	5.9	5.7	0.719
VAS: contentedness (mean)	base	76	18.7	19.6	0.767
	3 h	75	20.6	22.1	0.660
	δ	75	2.0	3.0	0.304
VAS: calmness (mean)	base	76	22.6	24.9	0.659
	3 h	75	23.0	23.4	0.812
	δ	75	0.4	-0.8	0.890
NVL: any effect	3h	75	-31.5	-38.3	0.743
NVL: bad effects	3h	75	-42.4	-43.1	0.439
NVL: good effects	3h	75	-40.2	-40.7	0.570
NVL: high	3h	75	-43.3	-41.6	0.204
NVL: rush	3h	75	-41.3	-43.6	0.270
NVL: like drug	3h	75	-16.8	-14.6	0.417
NVL: stimulated	3h	75	-39.4	-36.7	0.100
NVL: performance impaired	3h	75	-38.2	-35.6	0.152
NVL: performance improved	3h	75	-38.1	-41.1	0.629
NVL: willing to take again	3h	75	8.8	2.9	0.656
NVL: willing to pay for	3h	75	-39.3	-40.8	0.402
NVL: active-alert-energetic	3h	75	-35.1	-37.9	0.844
NVL: shaky/jittery	3h	75	-42.0	-36.0	0.337
NVL: euphoric	3h	75	-43.3	-38.7	0.158
NVL: irregular or racing heart	3h	75	-45.8	-44.7	0.153
NVL: talkative-friendly	3h	75	-39.1	-31.9	0.049
NVL: nauseated, queasy or sick to stomach	3h	75	-46.4	-46.5	0.393
NVL: nervous or anxious	3h	75	-42.9	-45.1	0.734
NVL: restless	3h	75	-36.2	-30.8	0.085
NVL: sluggish-lazy-fatigued	3h	75	-25.7	-23.6	0.487

Supplementary Table 14: Physiological and self-reported side effects following drug. (**Notes. Base = baseline; 3h = 3 hours after drug loading; δ = difference between the value 3 hours after drug loading and the baseline; N = number of observations; Plac. = Placebo group; Sulp. = Sulpiride group; Sign. = Significance of Mann-Whitney tests for differences.)**

Supplementary Note 1

Definition of reciprocal and mistake trials

To avoid ambiguity, we note the exact definition of Reciprocal and Mistake Trials used in the main text. Denoting *Backtransfer* as a variable for positive (1) or negative (-1) feedback from the Trustee, and *Change* as a variable for the relative change in investment from the previous trial, we defined Reciprocal and Mistake Trials as follows:

$$Reciprocal\,Trial = ((Backtransfer == 1\,\&\,Change > 0)\,|\,(Backtransfer == 1\,\&\,Investment == 10))\,|\,((Backtransfer == -1\,\&\,Change < 0)\,|\,(Backtransfer == -1\,\&\,Investment == 0)),$$

$$Mistake\,Trial = ((Backtransfer == 1\,\&\,Change < 0)\,|\,(Backtransfer == -1\,\&\,Change > 0))$$

Supplementary Note 2

Optimal behaviour in the repeated trust game

Optimal behaviour in the repeated trust game can be defined within various frameworks. Within the utility maximisation framework, a "rational" outcome-maximising agent would at each trial choose the investment that brings the highest expected value. When the trustee betrays the investor after an investment I, the investor ends up with an outcome of $10 - I$. When the trustee equalizes both players receive half of the overall gain. The outcome V in that case is:

$$V = \frac{I - 10 + 10 + 3 * I}{2} = 10 + I$$

If an agent has a subjective belief that the trustee will equalize with probability p, then the expected value of his investment I is:

$$EV(I) = p * (10 + I) + (1 - p) * (10 - I) = 10 + I(2p - 1),$$

which is a linear function of p. Meaning that as soon as p is believed to be above 0.5 the outcome maximising agent should give a ten and as soon as p is below 0.5 they should invest 0.

We note that in our model, an outcome-maximizing agent would have extremely high values of γ, which would also correspond to highly exploitative behaviour defined within the reinforcement learning framework.

Importantly, this outcome-maximizing agent does not consider the uncertainty about their estimate. On the other hand, a Bayesian perspective defines behaviour in this task as optimal given the investor's (participant's) subjective prior beliefs[3]. Update equations of the model are determined deterministically from the free parameters and describe Bayes optimal belief trajectories[4]. For example, if one's prior is that other people's behaviour is highly volatile, then it is optimal to adjust your belief after each feedback, whereas if one expects individuals to behave consistently, one should form an opinion quickly. With this, the Bayesian framework can describe subjectively optimal behaviours that are objectively maladaptive[4].

Supplementary Figures

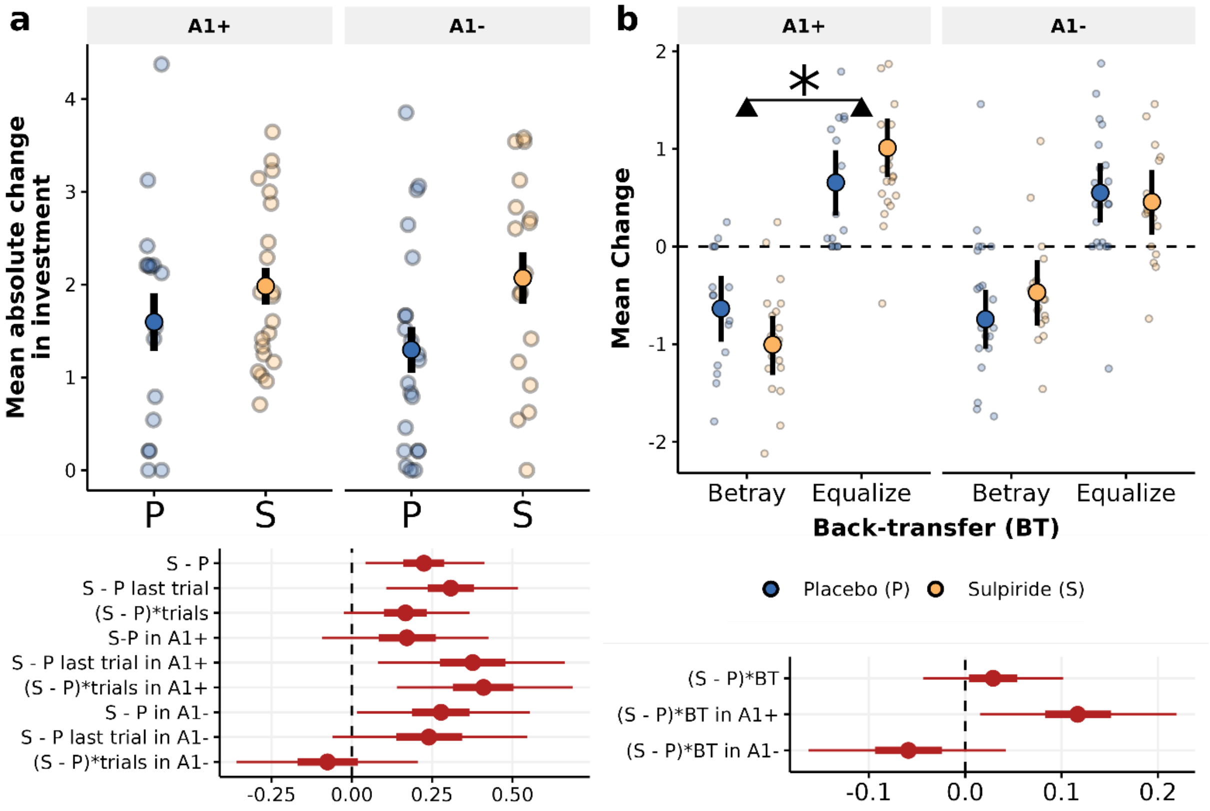

Supplementary Fig. 1 | Effects of sulpiride on behaviour in the repeated Trust game. a, Mean and standard errors of absolute change of investment from one trial to the next for both treatment and genotype groups. 95% CrI of effect sizes show a main effect of sulpiride, no credible evidence for an interaction effect of treatment and genotype, but a differential effect of sulpiride on the slope of two genotype groups. Sample sizes in A1+ group, n=17 placebo, n=21 sulpiride, and in A1- group n=21 placebo and n=17 sulpiride. **b**, Mean and 95% CrI of relative change in investment following positive and negative feedback, for both genotype groups separately, predicted from a Bayesian multilevel model including Genotype as a predictor. Dots are raw means for each participant. Effect sizes below. The 95% CrI of the interaction effect of Drug and Back-transfer was above zero only in the A1+ group. We found no credible evidence for an interaction effect of sulpiride and Back-transfer (b = 0.089, 95% CrI [-0.135, 0.314], P(b<0) = 0.217, d = 0.029, 95% CrI [-0.044, 0.102]), but observed a three-way interaction effect, with the Genotype variable (b = -0.545, 95% CrI [-0.987, -0.103], P(b>0) = 0.007, d = -0.176, 95% CrI [-0.319, -0.034]). In the A1+ group, participants administered the D2 antagonist tended to increase their investment following positive, and decrease their investment following negative, responses from the trustees (b = 0.361, 95% CrI [0.048, 0.68], P(b<0) = 0.013, d = 0.116, 95% CrI [0.016, 0.219]). We found no credible evidence of a difference between sulpiride and placebo administration in A2 homozygotes (b = -0.179, 95% CrI [-0.502, 0.131] , P(b>0) = 0.127, d = -0.057, 95% CrI [-0.159, 0.042]). Sample sizes as in **a**.

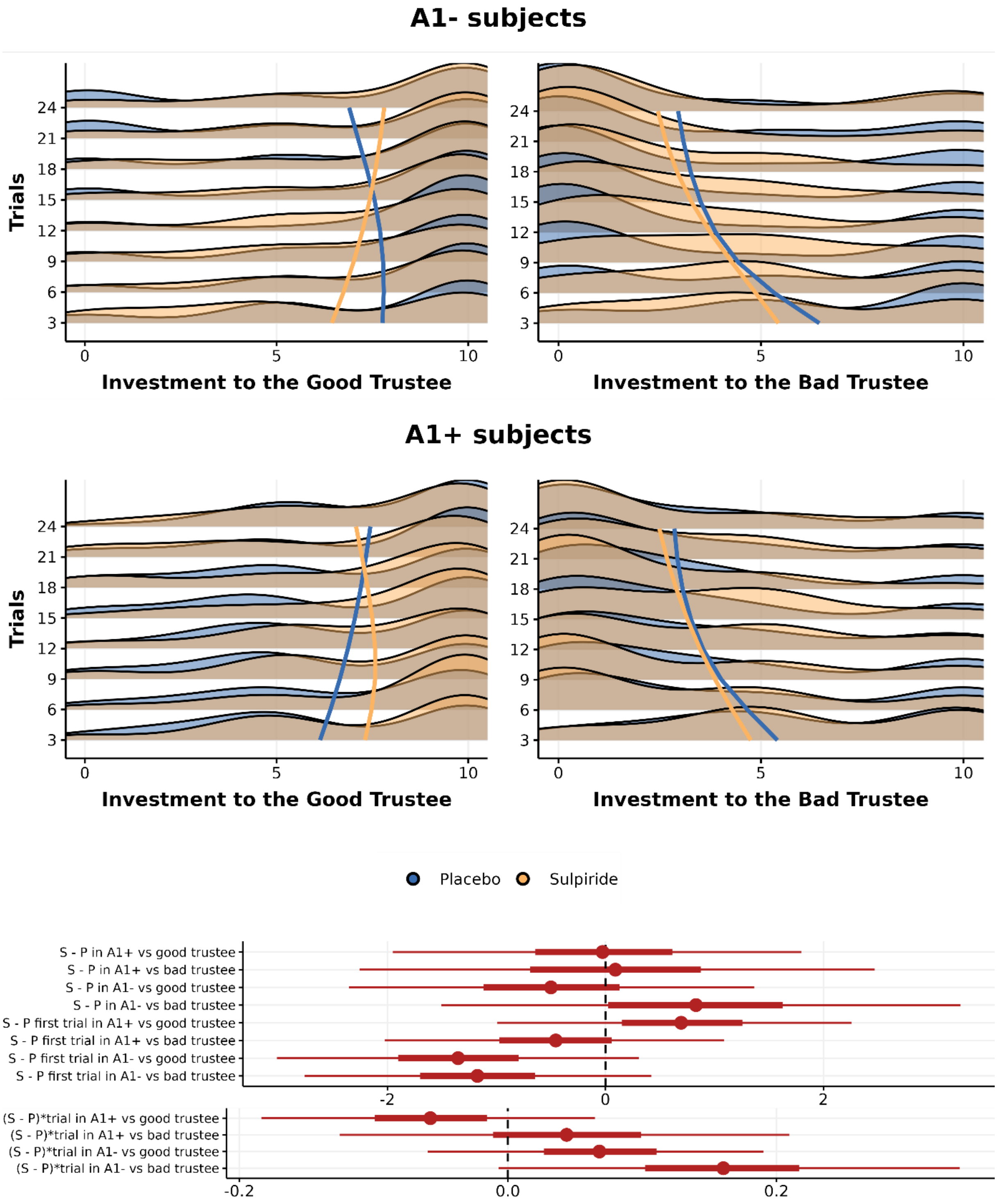

Supplementary Fig. 2 | Effects of sulpiride on average investments in the repeated Trust game. Density plots of investments for placebo and sulpiride group overlayed and grouped within the trustee and genotype (binned across 3 trials for clarity). Lines are means of investments predicted from a multilevel Bayesian model that included Trial, Genotype, Trustee and Treatment as fixed and Trustee and Trial as participant-level random effects. Effect sizes are shown below for average investments, differences in the first trial (initial trustworthiness) as well as for the slope with means (and 50% and 95% CrI).

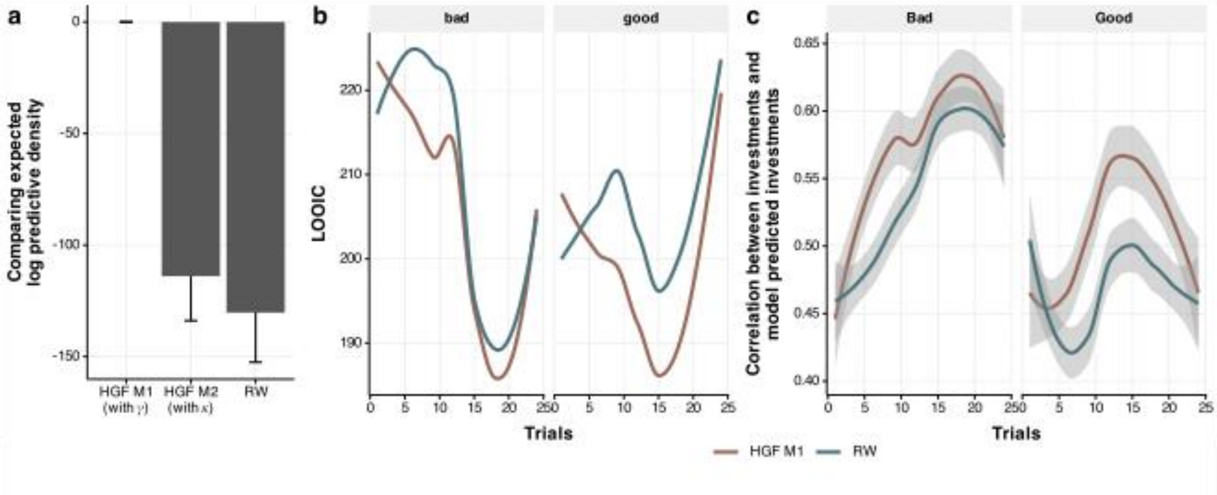

Supplementary Fig. 3 | Model comparison. a, Comparing the HGF model with all four parameters (ω, μ_0, η, γ) to an HGF model that includes a coupling parameter κ, but does not include γ and to an RW model. On the y-axis is the relative difference in the predictive density (on a log scale) together with the standard errors (obtained by the loo_compare function of the loo package in R). Model comparison done for all 76 participants. **b**, The HGF model with γ and the RW model compared with the leave-one-out-information criterion across trials and trustees (25 trials per trustee), lower values imply a better fit. **c**, Same two models compared based on the correlations of actual investments and investments predicted by the model (higher values indicate a better fit). Lines depicted means per model and standard errors across all participants (n = 76).

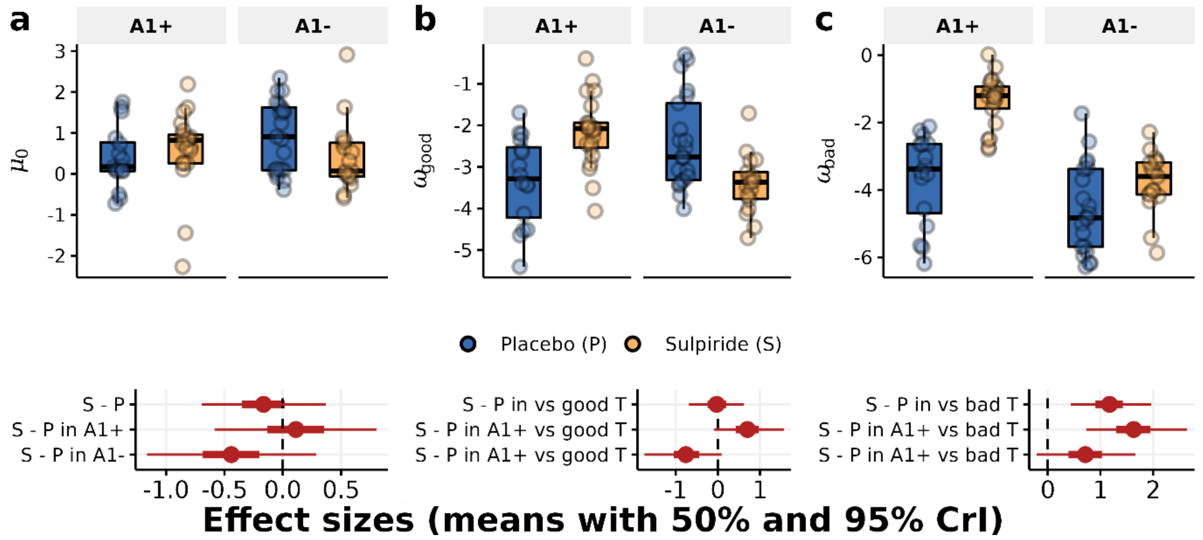

Supplementary Fig. 4 | Sulpiride's effects on model parameters. a, Effect of sulpiride on initial trustworthiness belief. **b**, Effects of sulpiride on belief volatility when playing against the good trustee. **c**, Effects of sulpiride on belief volatility when playing against the bad trustee. Below are mean effect sizes with 50% and 95% CrI. Sample sizes in A1+ group, n=17 placebo, n=21 sulpiride, and in A1- group n=21 placebo and n=17 sulpiride. Boxplots with centre line as medians, box bounds 25th and 75th percentiles, and whiskers terminating at maxima/minima (a distance of 1.5 times the IQR away from the 25th and 75th percentiles).

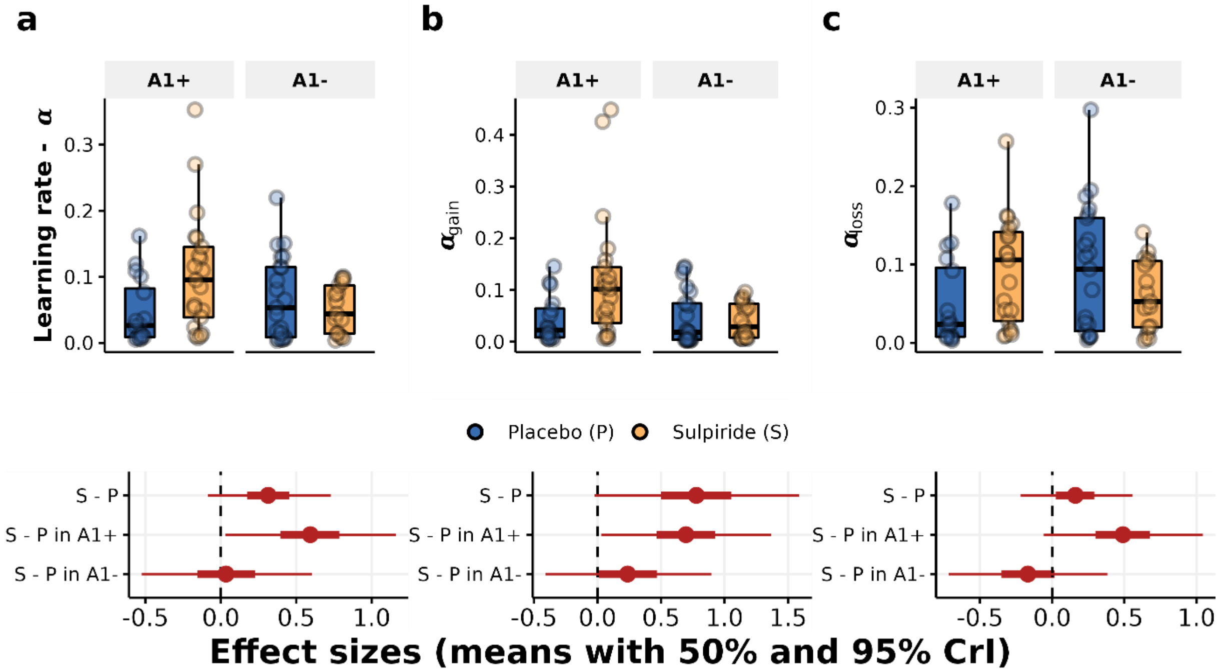

Supplementary Fig. 5 | Sulpiride effects on the RW model's learning rate. a, Average learning rate across trials. **b**, Learning rate for positive outcomes. **c**, Learning rate for negative outcomes. Below are mean effect sizes with 50% and 95% CrI. Sample sizes in A1+ group, n=17 placebo, n=21 sulpiride, and in A1- group n=21 placebo and n=17 sulpiride. Boxplots with centre line as medians, box bounds 25th and 75th percentiles, and whiskers terminating at maxima/minima (a distance of 1.5 times the IQR away from the 25th and 75th percentiles).

Supplementary References

1. Naef, M. *et al.* Effects of dopamine D2/D3 receptor antagonism on human planning and spatial working memory. *Transl. Psychiatry* **7**, e1107 (2017).

2. Eisenegger, C. *et al.* Role of Dopamine D2 Receptors in Human Reinforcement Learning. *Neuropsychopharmacology* **39**, 2366–2375 (2014).

3. Daunizeau, J. *et al.* Observing the observer (I): Meta-bayesian models of learning and decision-making. *PLoS One* **5**, (2010).

4. Mathys, C. D., Daunizeau, J., Friston, K. J. & Stephan, K. E. A bayesian foundation for individual learning under uncertainty. *Front. Hum. Neurosci.* **5**, 39 (2011).
