# Supplementary material for: Blocking D2/D3 dopamine receptors in male participants increases volatility of beliefs when learning to trust others
Source: Nat Commun. 2023 Jul 8;14:4049. doi: 10.1038/s41467-023-39823-5 (PMC10329681; doi:10.1038/s41467-023-39823-5)
Supplement: Supplementary file 3 — Reporting Summary [file 41467_2023_39823_MOESM3_ESM.pdf]

nature portfolio

Corresponding author(s): Nace Mikus

Last updated by author(s): 29/3/23

Reporting Summary

Nature Portfolio wishes to improve the reproducibility of the work that we publish. This form provides structure for consistency and transparency in reporting. For further information on Nature Portfolio policies, see our Editorial Policies and the Editorial Policy Checklist.

Statistics

For all statistical analyses, confirm that the following items are present in the figure legend, table legend, main text, or Methods section.

n/a	Confirmed	
☐	☒	The exact sample size (*n*) for each experimental group/condition, given as a discrete number and unit of measurement
☐	☒	A statement on whether measurements were taken from distinct samples or whether the same sample was measured repeatedly
☐	☒	The statistical test(s) used AND whether they are one- or two-sided<br>*Only common tests should be described solely by name; describe more complex techniques in the Methods section.*
☐	☒	A description of all covariates tested
☐	☒	A description of any assumptions or corrections, such as tests of normality and adjustment for multiple comparisons
☐	☒	A full description of the statistical parameters including central tendency (e.g. means) or other basic estimates (e.g. regression coefficient) AND variation (e.g. standard deviation) or associated estimates of uncertainty (e.g. confidence intervals)
☐	☒	For null hypothesis testing, the test statistic (e.g. *F*, *t*, *r*) with confidence intervals, effect sizes, degrees of freedom and *P* value noted<br>*Give P values as exact values whenever suitable.*
☐	☒	For Bayesian analysis, information on the choice of priors and Markov chain Monte Carlo settings
☐	☒	For hierarchical and complex designs, identification of the appropriate level for tests and full reporting of outcomes
☐	☒	Estimates of effect sizes (e.g. Cohen's *d*, Pearson's *r*), indicating how they were calculated

Our web collection on statistics for biologists contains articles on many of the points above.

Software and code

Policy information about availability of computer code

Data collection	Visual Basic 2008/2010
Data analysis	R (v 4.1.3 and older), RStudio (v 1.4.1717 and older), computational modelling done in Stan (v 2.21.0 and older) with custom scripts. All code used in the analysis available online (https://github.com/nacemikus/belief-volatility-da-trustgame.git)

For manuscripts utilizing custom algorithms or software that are central to the research but not yet described in published literature, software must be made available to editors and reviewers. We strongly encourage code deposition in a community repository (e.g. GitHub). See the Nature Portfolio guidelines for submitting code & software for further information.

Data

Policy information about availability of data

All manuscripts must include a data availability statement. This statement should provide the following information, where applicable:

- Accession codes, unique identifiers, or web links for publicly available datasets
- A description of any restrictions on data availability
- For clinical datasets or third party data, please ensure that the statement adheres to our policy

All data used in the MS available online (https://doi.org/10.5281/zenodo.7779029)

Human research participants

Policy information about studies involving human research participants and Sex and Gender in Research.

Reporting on sex and gender	Data was collected from male participants only. Information on participants used to preselect them was available through the volunteer bank (see below).
Population characteristics	Participants were stratified based on their DA D2 receptor Taq1A genotype, with one group consisting of individuals carrying one or two copies of the A1 allele and the other group consisting of A2 allele homozygotes. All were right-handed, male, European or North American caucasians, with an age range of 19–44 years (mean = 32.1).
Recruitment	Participants were recruited from the Cambridge BioResource, a large community-based panel of volunteers that agreed to take part in research linking genotype with phenotype (http://www.cambridgebioresource.org.uk). Prior to genotyping, adetailed medical history was obtained from all participants to assess their mental and physical health. This revealed no history of neurological disease or psychiatric disorders. All volunteers were naive to the task and none had participated in previous psychoactive drug studies. However, we cannot exclude other participation biases (such as those related to sociodemographic factors), which might affect the degree to which monetary rewards in incentivized task affect behaviour.
Ethics oversight	National Research Ethics Committee of Hertfordshire (11/EE/0111)

Note that full information on the approval of the study protocol must also be provided in the manuscript.

Field-specific reporting

Please select the one below that is the best fit for your research. If you are not sure, read the appropriate sections before making your selection.

☒ Life sciences ☐ Behavioural & social sciences ☐ Ecological, evolutionary & environmental sciences

For a reference copy of the document with all sections, see nature.com/documents/nr-reporting-summary-flat.pdf

Life sciences study design

All studies must disclose on these points even when the disclosure is negative.

Sample size	A sample size of 40 participants per drug group (80 in sum) and 20 per drug/genotype group was planned. 78 participants ended up participating in the study. The sample size was chosen based on previous research with the same (or similar) compounds (Dodds et al., 2009 DOI 10.1007/s00213-009-1634-0) and genotypes (Jocham et al., 2009 10.1523/JNEUROSCI.5195-08.2009)
Data exclusions	Two participants were excluded from the analysis: one felt uncomfortable in the room, and one did not sufficiently understand the instructions of the social interaction tasks. This led to the following group distributions: 17 A1 allele carriers received placebo, and 21 received sulpiride, and 21 A2 homozygotes received placebo, and 17 received sulpiride. One further individual was excluded due to missing data on the working memory task.
Replication	No direct replication of the findings was conducted (since this would entail another pharmacological study of this size). We aimed to increase the robustness and reproducibility of results by reporting both frequentist and Bayesian model summaries and by ensuring that the effect we find is present across similar behavioural outcomes (for example, reciprocity was measured as a count of reciprocal trials, as well as the size of the investment change following positive or negative feedback).
Randomization	Participants were randomly assigned to receive the active compound or placebo, while ensuring that the resulting four groups of participants were all matched for age and BMI with genotype groups of similar size.
Blinding	The study was double blinded.

Reporting for specific materials, systems and methods

We require information from authors about some types of materials, experimental systems and methods used in many studies. Here, indicate whether each material, system or method listed is relevant to your study. If you are not sure if a list item applies to your research, read the appropriate section before selecting a response.

Materials & experimental systems

n/a	Involved in the study
☒	☐ Antibodies
☒	☐ Eukaryotic cell lines
☒	☐ Palaeontology and archaeology
☒	☐ Animals and other organisms
☒	☐ Clinical data
☒	☐ Dual use research of concern

Methods

n/a	Involved in the study
☒	☐ ChIP-seq
☒	☐ Flow cytometry
☒	☐ MRI-based neuroimaging
